# Supplementary material for: Reorientation behavior in the helical motility of light-responsive spiral droplets
Source: Nat Commun. 2019 Nov 20;10:5238. doi: 10.1038/s41467-019-13201-6 (PMC6868138; doi:10.1038/s41467-019-13201-6)
Supplement: Supplementary file 1 — Supplementary Information [file 41467_2019_13201_MOESM1_ESM.pdf]

Reorientation behaviour in the helical motility of light-responsive spiral droplets

Federico Lancia,[1] Takaki Yamamoto,[2] Alexander Ryabchun,[1] Tadatsugu Yamaguchi,[1] Masaki Sano,[3,4]* and Nathalie Katsonis[1]*

[1] Bio-inspired and Smart Materials, MESA+ Institute for Nanotechnology, University of Twente, PO Box 207, Enschede 7500AE, The Netherlands.

[2] Laboratory of Physical Biology, RIKEN Center for Biosystems Dynamics Research, Kobe 650-0047, Japan.

[3] Universal Biology Institute, The University of Tokyo, 7-3-1 Hongo, Bunkyo-ku, Tokyo 113-0033, Japan.

[4] Institute for Natural Sciences, Shanghai Jao Tong University, No. 800 Dongchuan Rd, Minhang District, Shanghai 200240, China

* Correspondence to: sano@phys.s.u-tokyo.ac.jp ; n.h.katsonis@utwente.nl

Supplementary Information

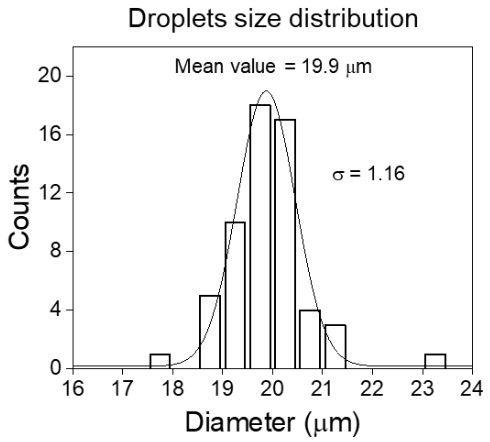

Supplementary Figure 1. Polydispersity of the cholesteric droplets produced by injection in water.

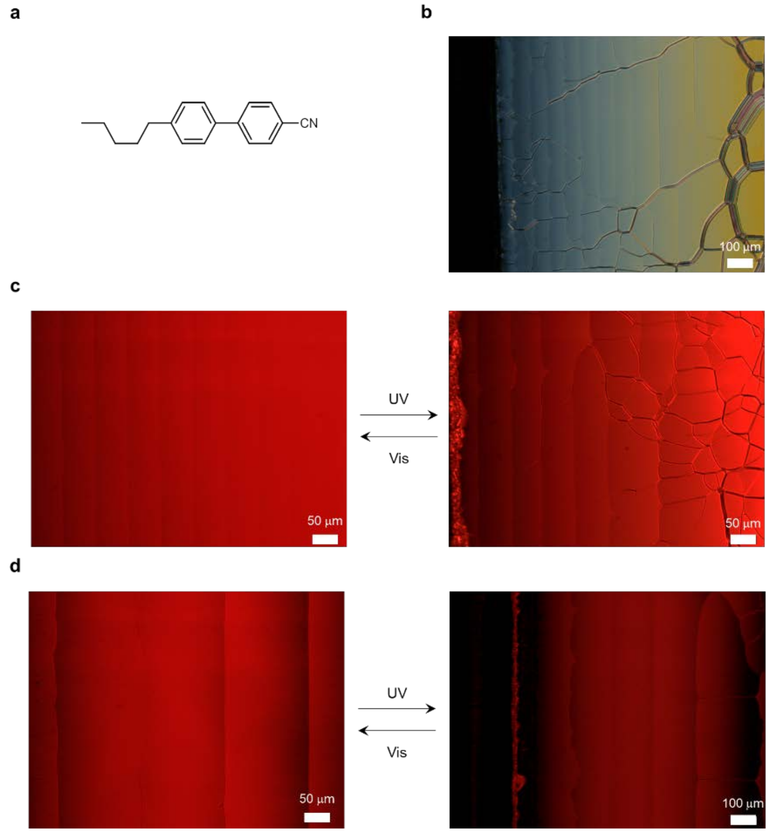

Supplementary Figure 2. Characterization of the cholesteric liquid crystals used for the production of the droplets. a, Nematic liquid crystal 5CB used as achiral host in all the compositions of cholesteric liquid crystals. **b**, **c**, **d**, Polarized optical microscopy images of wedge cells containing cholesteric liquid crystals doped with CB15(10 wt %), Ph-**m** (0.68 wt %) and Me-**m** (0.38 wt %), respectively. The Grandjean-Cano lines are visible and were used to measure the pitch of the cholesteric liquid crystal. For **c** and **d** the image on the right corresponds to the liquid crystal at the photo-stationary state (inverted handedness), for which the pitch is also measured.

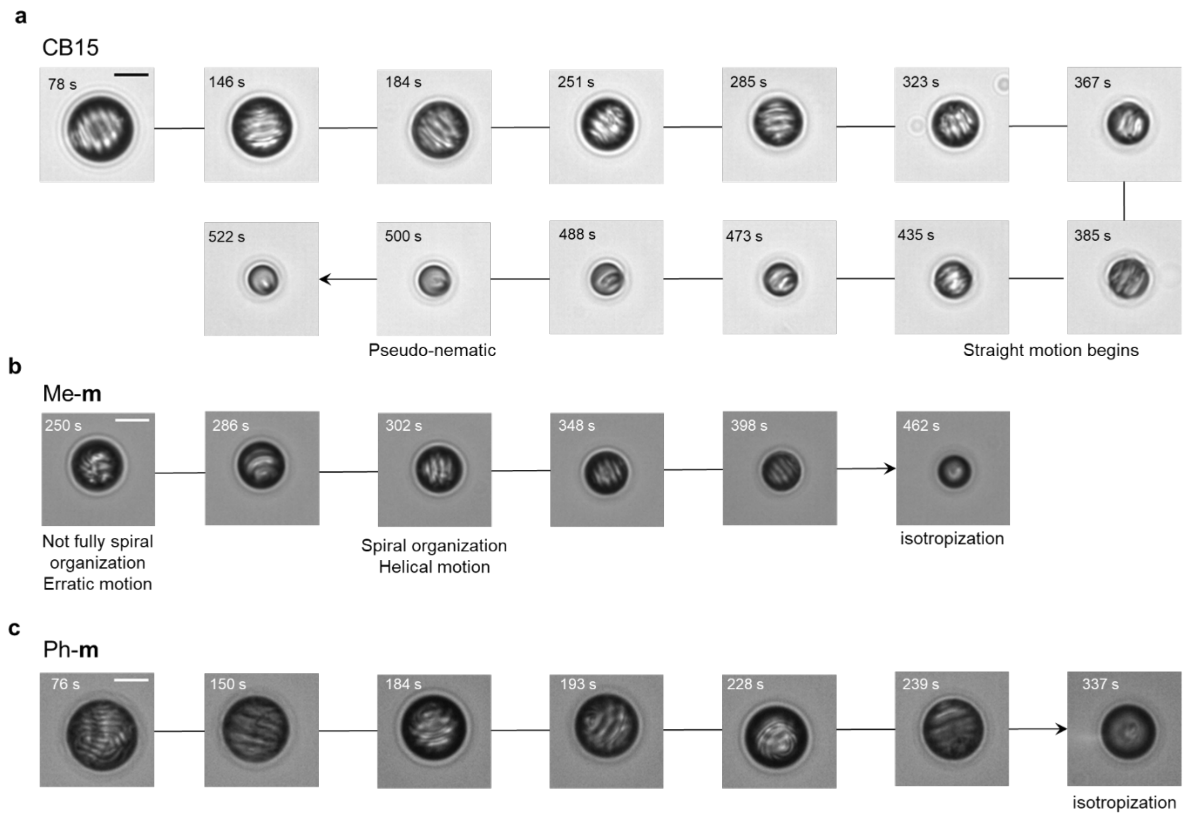

Supplementary Figure 3. Evolution of the droplet in time, for different chiral dopants. a, Droplets doped with CB15 initially display a well-defined spiral distribution of the director field. In time, the number of spiral disclination lines decreases and eventually (after ~ 500 s), the chirality of the droplet is not expressed at the structural level anymore, i.e. the droplet can be considered pseudo-nematic. **b**, Droplets doped with Me-**m** need time to organize into the spiral configuration, after what they start swimming along helical trajectories (here after ~ 300 s) and eventually the droplets become isotropic. **c**, Droplets doped with Ph-**m** swim helically after being created and they maintain a clear helical trajectory until isotropization occurs at 337s. The occurrence of isotropization for Ph-**m** and Me-**m** doped cholesteric droplets suggests that the dopant is concentrating in the droplet over time, in contrast to what happens when CB15 is used as a dopant. Scales bar are 10 μm.

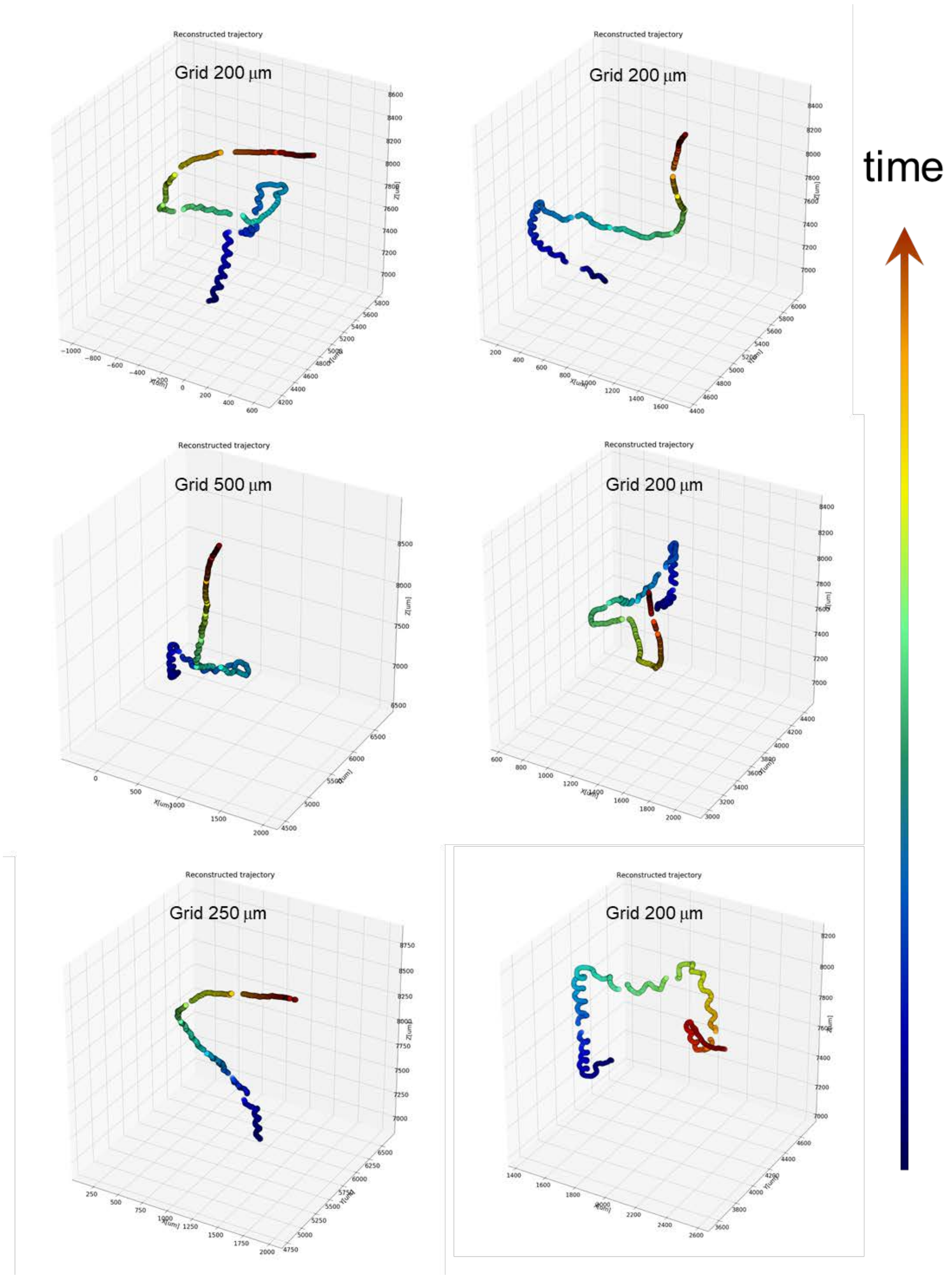

Supplementary Figure 4. Three dimensional trajectories for droplets doped with CB15. Notably the motion is helical right after injection of the droplet in the aqueous medium. The chirality confinement ratio N decreases over time until eventually the droplets loses its chiral configuration and propel straight. The top and bottom of the chambers are always at z values 8100 and 7000 µm, respectively.

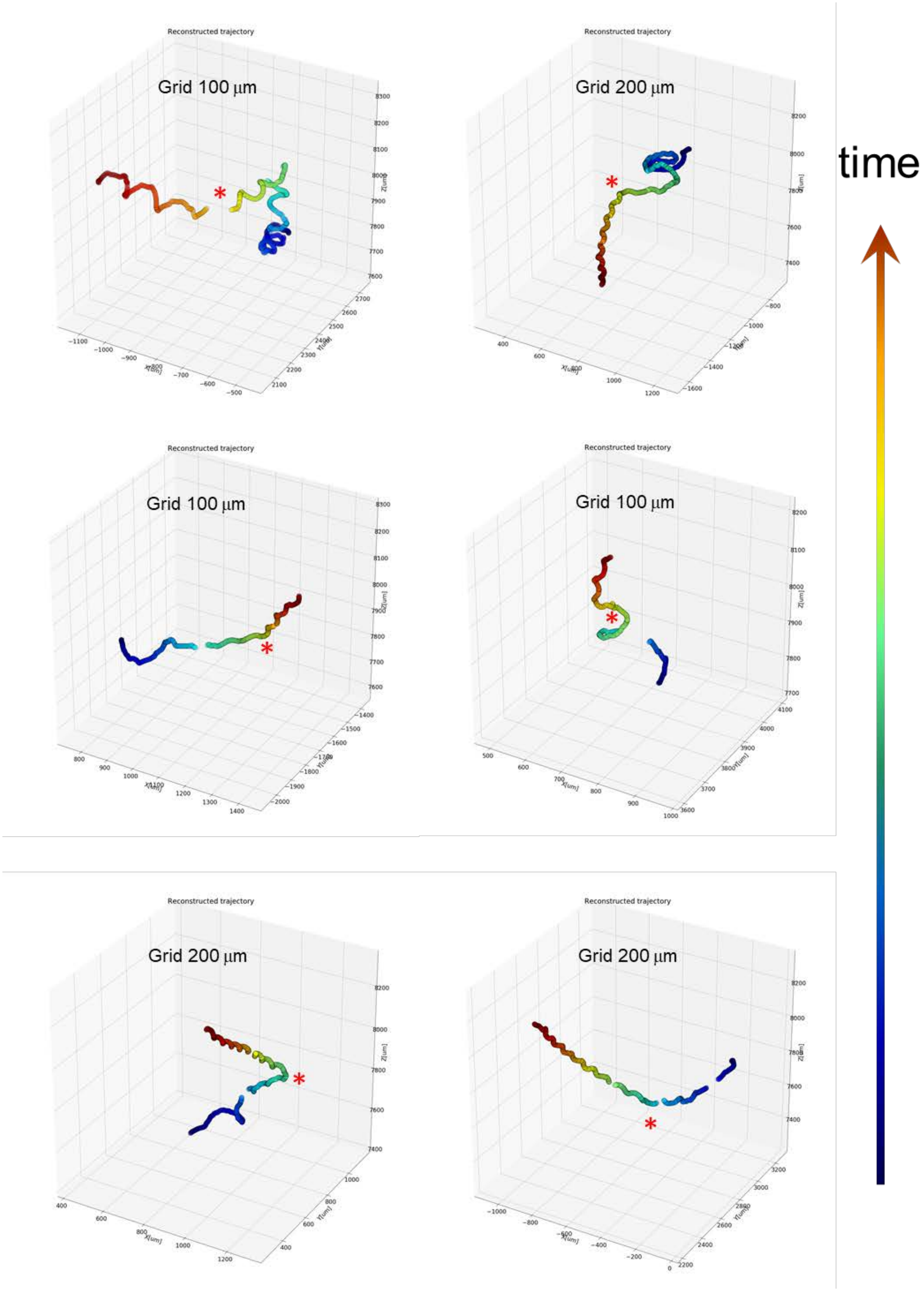

Supplementary Figure 5. Three dimensional trajectories with abrupt helix inversion and directional change . The droplets are doped with Me-m. The red asterisks mark the switching event. The top and bottom of the chambers are always at z values 8100 and 7000 μm, respectively.

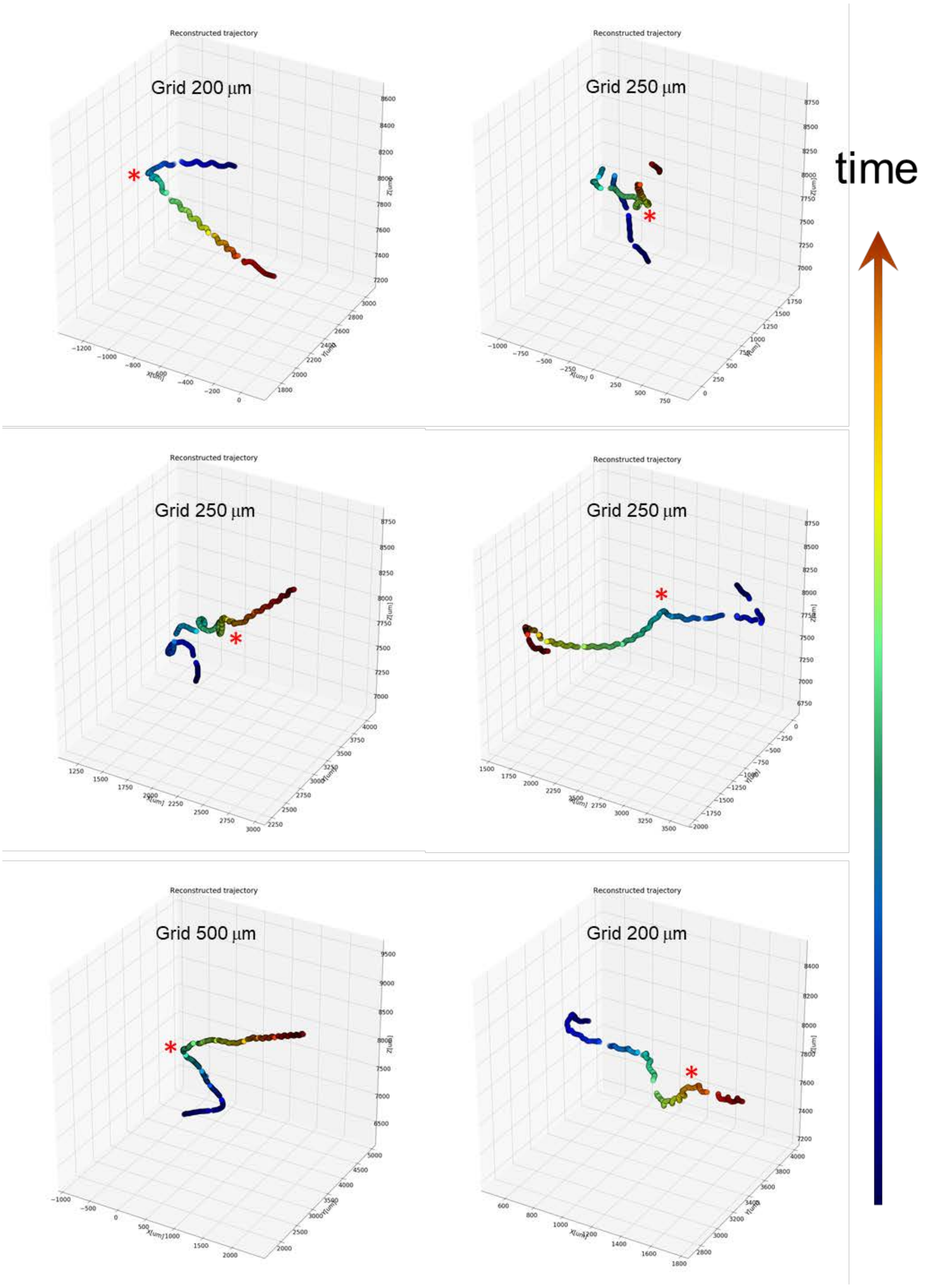

Supplementary Figure 6. Three dimensional trajectories with abrupt helix inversion and directional change . The droplets are doped with Me-m. The red asterisks mark the switching event. The top and bottom of the chambers are always at z values 8100 and 7000 μm, respectively.

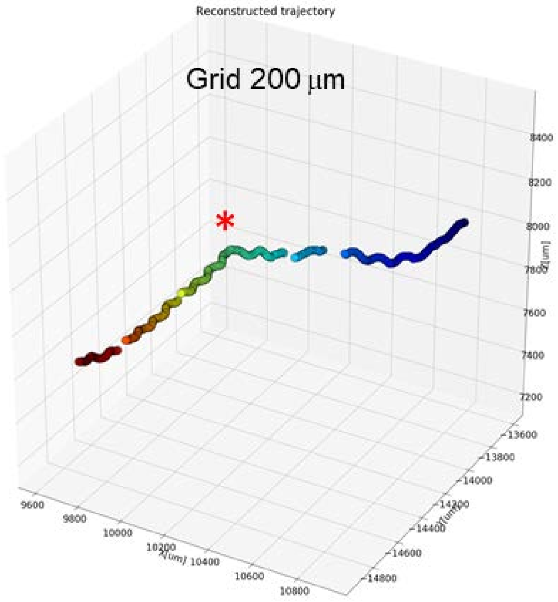

Supplementary Figure 7. Three dimensional trajectory with abrupt helix inversion and directional change . The droplet is doped with Me-m. The red asterisks mark the switching event. The top and bottom of the chambers are always at z values 8100 and 7000 µm, respectively.

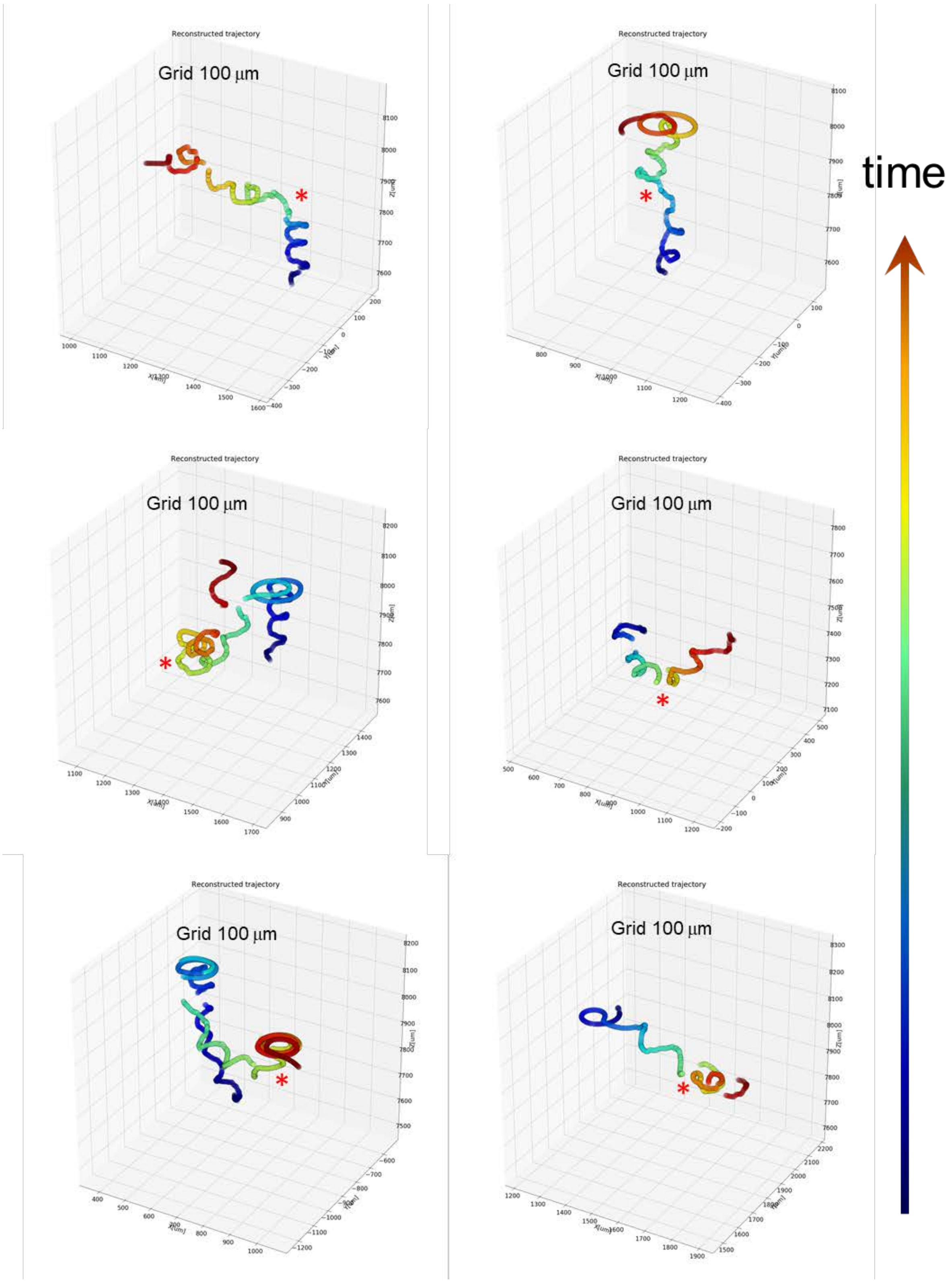

Supplementary Figure 8. Three dimensional trajectories with abrupt helix inversion and directional change. The droplets are doped with Ph-m. The red asterisks mark the switching event. The grid is 100 μm × 100 μm × 100 μm. The top and bottom of the chambers are always at z values 8100 and 7000 μm, respectively.

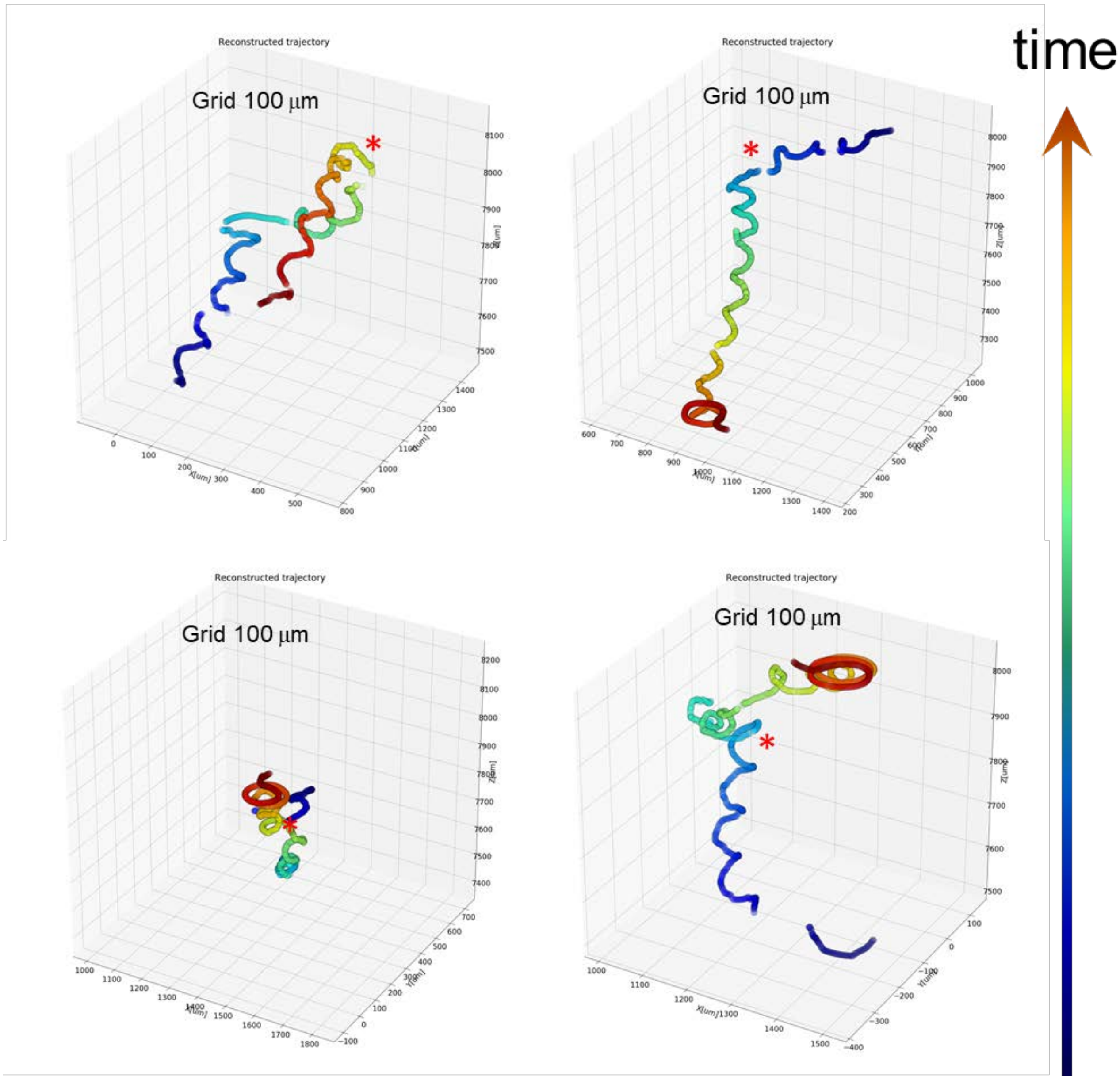

Supplementary Figure 9. Three dimensional trajectories with abrupt helix inversion and directional change . The droplets are doped with Ph-m. The red asterisks mark the switching event. The grid is 100 μm × 100 μm × 100 μm. The top and bottom of the chambers are always at z values 8100 and 7000 μm, respectively..

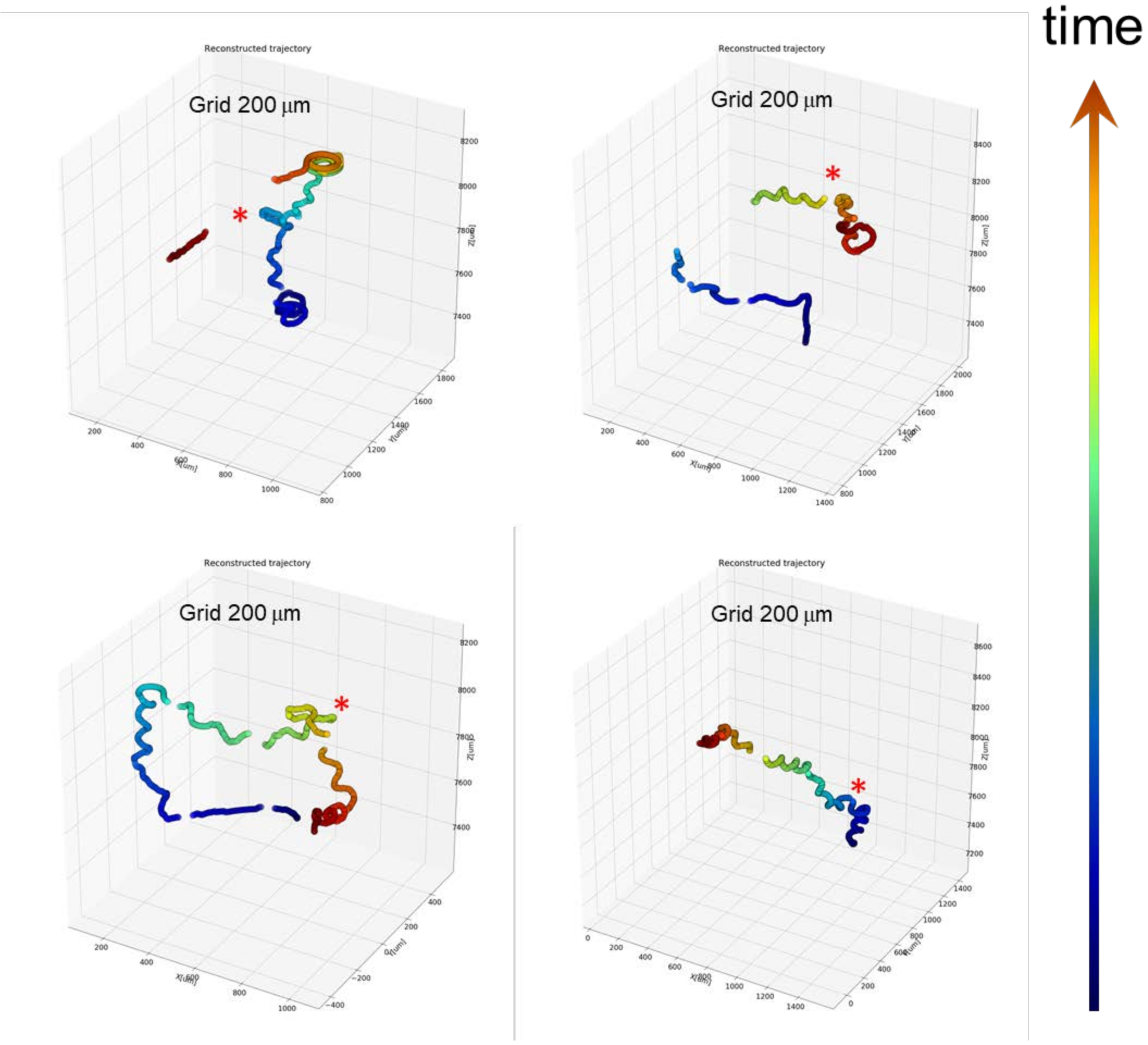

Supplementary Figure 10. Three dimensional trajectories with abrupt helix inversion and directional change . The droplets are doped with Ph-m. The red asterisks mark the switching event. The grid is 200 μm × 200 μm × 200 μm. The top and bottom of the chambers are always at z values 8100 and 7000 μm, respectively..

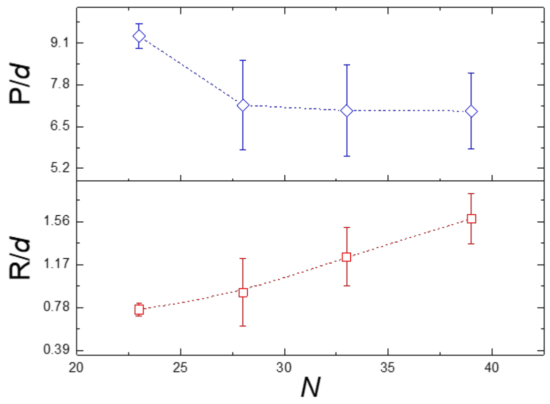

Supplementary Figure 11. Evolution of the pitch and radius of the helical trajectory with increasing values of chirality confinement *N*. The relative radius (R/d, where d is the diameter of the droplet) increase with the increase in chirality confinement ratio *N*. The relative pitch (P/d ,where d is diameter of the droplet) decreases with an increase of *N*. The error bars represent the standard deviation.
