# Supplementary material for: Reorientation behavior in the helical motility of light-responsive spiral droplets
Source: Nat Commun. 2019 Nov 20;10:5238. doi: 10.1038/s41467-019-13201-6 (PMC6868138; doi:10.1038/s41467-019-13201-6)
Supplement: Supplementary file 3 — Description of Additional Supplementary Files [file 41467_2019_13201_MOESM3_ESM.pdf]

Description of Additional Supplementary Files

File Name: Supplementary Movie 1
Description: **Helical motion of a spiral droplet.** The movie shows the motion of a liquid crystal droplet doped with molecular motor Ph-**m**. The movie is accelerated 1.5 times

File Name: Supplementary Movie 2
Description: **Reorientation of a spiral droplet.** A droplet doped with Ph-**m** follows a left-handed helical trajectory before the chirality of the spiral droplet is inverted, by irradiation with UV light (λ = 365 nm, 300 $mW.cm^{-2}$). The helix inversion is accompanied by a significant reorientation. The movie is accelerated 1.5 times.

File Name: Supplementary Movie 3
Description: **Precession of the droplet along the trajectory.** The movie shows a chiral droplet swimming vertically. The axis of the chiral droplet maintains a constant (precession) angle with respect to the direction of the movement.

File Name: Supplementary Movie 4
Description: **No significant reorientation occurs upon gradual helix inversion.** A droplet doped with Me-**m** is irradiated with low power UV light (λ = 365 nm, 50 $mW.cm^{-2}$). The spiral droplet gradually unwinds into a compensated nematic before rewinding into spiral organization with opposite handedness. As the chiral character of the motion is lost, the change in propagation direction is not significant. The movie is accelerated 1.5 times.
